# Contributions to the Medical Flora of Nashville, Tenn.

**Published:** 1860-12

**Authors:** 


Contributions to the Medical Flora of Nashville, Tenn. By George S. Blackie, M. D., Prof. of Botany and Natural History in the University of Nashville.

This little Pamphlet of 23 pages, was designed to call the attention of the practitioners of Medicine in the vicinity of Nashville, to the numerous and important indiginous articles found growing in their own woods, fields and road-sides. It is well written, and will serve a useful purpose.

